# Directed retreat and navigational mechanisms in trail following *Formica obscuripes*

**DOI:** 10.3758/s13420-023-00604-1

**Published:** 2023-09-26

**Authors:** Cody A. Freas, Marcia L. Spetch

**Affiliations:** 1 Department of Psychology, University of Alberta, Edmonton, Alberta Canada; 2 School of Natural Sciences, Macquarie University, Sydney, NSW 2113 Australia

**Keywords:** Orientation, Thatching ants, Pheromone, Reassurance cues, Multi-modal cues, Cue interactions

## Abstract

Ant species exhibit behavioural commonalities when solving navigational challenges for successful orientation and to reach goal locations. These behaviours rely on a shared toolbox of navigational strategies that guide individuals under an array of motivational contexts. The mechanisms that support these behaviours, however, are tuned to each species’ habitat and ecology with some exhibiting unique navigational behaviours. This leads to clear differences in how ant navigators rely on this shared toolbox to reach goals. Species with hybrid foraging structures, which navigate partially upon a pheromone-marked column, express distinct differences in their toolbox, compared to solitary foragers. Here, we explore the navigational abilities of the Western Thatching ant (*Formica* *obscuripes*), a hybrid foraging species whose navigational mechanisms have not been studied. We characterise their reliance on both the visual panorama and a path integrator for orientation, with the pheromone’s presence acting as a non-directional reassurance cue, promoting continued orientation based on other strategies. This species also displays backtracking behaviour, which occurs with a combination of unfamiliar terrestrial cues and the absence of the pheromone, thus operating based upon a combination of the individual mechanisms observed in solitarily and socially foraging species. We also characterise a new form of goalless orientation in these ants, an initial retreating behaviour that is modulated by the forager’s path integration system. The behaviour directs disturbed inbound foragers back along their outbound path for a short distance before recovering and reorienting back to the nest.

**Supplementary Information:**

The online version contains supplementary material available at 10.3758/s13420-023-00604-1.

## Introduction

Extensive research by Ken Cheng and colleagues on navigation in ants has highlighted the value of a comparative ecological approach in which mechanisms are studied in the context of their function in the natural environment (e.g., Bühlmann et al., 2011, 2020; Cheng et al., 2009, 2012, 2014; Cheng, 2022, 2023; Freas & Cheng, 2022; Freas et al., 2018, 2019a, b, c; Schultheiss et al., 2016). Comparisons between ant species have revealed many similarities that exist across a wide range of environments and foraging ecologies, with the presence of a common underlying navigational toolkit of concurrently operating strategies (Bühlmann et al., 2011; Cheng et al., 2009; Freas & Spetch, 2023; Wehner, 2020). Of these, the most frequently observed across ant species consists of an updating vector maintained by the path integration system reliant on a celestial compass (Collett & Collett, 2000; Wehner et al., 1996; Wehner, 2020). When visual terrestrial cues are available, ants often also use these visual landmarks, by learning and retaining panoramic views at the nest and along foraging routes for later comparison when orienting (Cheng et al., 2009; Freas & Cheng, 2018; Freas & Spetch, 2019; Narendra et al., 2007; Schultheiss et al., 2016; Wystrach et al., 2011; Zeil, 2012; Zeil & Fleischmann, 2019). While these commonalities across species are interesting, just as intriguing are mechanistic differences expressed by various ant species to solve similar spatial challenges. Such differences can arise from the cue availability in the local environment but are also closely tied with the foraging ecology of each individual species (Bühlmann et al., 2011; Cheng et al., 2014; Freas at al., 2019a, b, c; Schwarz & Cheng, 2010).

Within ants, many well-studied species forage solitarily and must navigate alone, yet trail following ants rely upon many of the same navigational mechanisms with the additional complexity of the pheromone trail also helping to direct movement. Much of what we know regarding the strategies of navigating ants is based on solitarily foraging species that rely heavily on the visual cues of the celestial compass and the surrounding panorama (the 360º scene given ants see in a ~300º field of view) to navigate (Cheng et al., 2009; Freas et al., 2021; Narendra et al., 2017; Warrant & Dacke, 2016; Wehner, 2020; Wystrach et al., 2013). However, there has been parallel interest focused on how trail following ants integrate both individual and communal types of information to navigate (Almeida et al., 2018; Aron et al., 1993; Czaczkes et al., 2019, 2022; Freas & Spetch, 2021; Grüter et al., 2011; Jones et al., 2019; Minoura et al., 2016; Middleton et al., 2018).

Recent navigational work in a Sonoran Desert harvester ant, *Veromesser pergandei*, presents a demonstration of the underlying mechanistic differences observed between socially and individually foraging species*. V.* *pergandei* foragers exhibit a hybrid foraging structure, termed a “column and fan” (Plowes et al., 2013, 2014). These ants begin their foraging journey socially, along a pheromone-marked column before individuals exit the pheromone and fanning out several meters to forage alone. Foragers maintain a path-integration-derived vector both in the column and the fan (Freas et al., 2019a,b). Once food is collected, foragers return first to the end of the column (column head) using a path-integration-derived local vector (Freas et al., 2020), then shift their headings to follow their global vector along the pheromone column to the nest (see also Flanagan et al., 2013, for potential evidence the phenomena may also be present in trunk trail systems). This pheromone mechanism mediating inhibition of part of the path integration system is in stark contrast to local vectors in solitarily foraging species, which are instead mediated by familiar views along the route (Collett et al., 1998; Webb, 2019).

Local vectors represents but one of multiple mechanistic differences underlying similar path integration-linked behaviours, including maintaining orientation, backtracking and partial vector suppression, all of which are mediated in *V.* *pergandei* by the presence of the pheromone rather than panorama views as is the case for solitary foraging ants (Collett et al., 1998; Freas et al., 2019a, b, c, 2020; Freas & Spetch, 2021; Plowes et al., 2019; Wystrach et al., 2013). In fact, with regard to view-based navigation, *V.* *pergandei* shows no evidence of using view alignment of the panorama to orient, despite living in a visually cluttered environment where sympatric solitarily foraging ant species actively rely on these same views to home (Freas et al., 2019a, b, c, 2021).

**Fig. 1:**
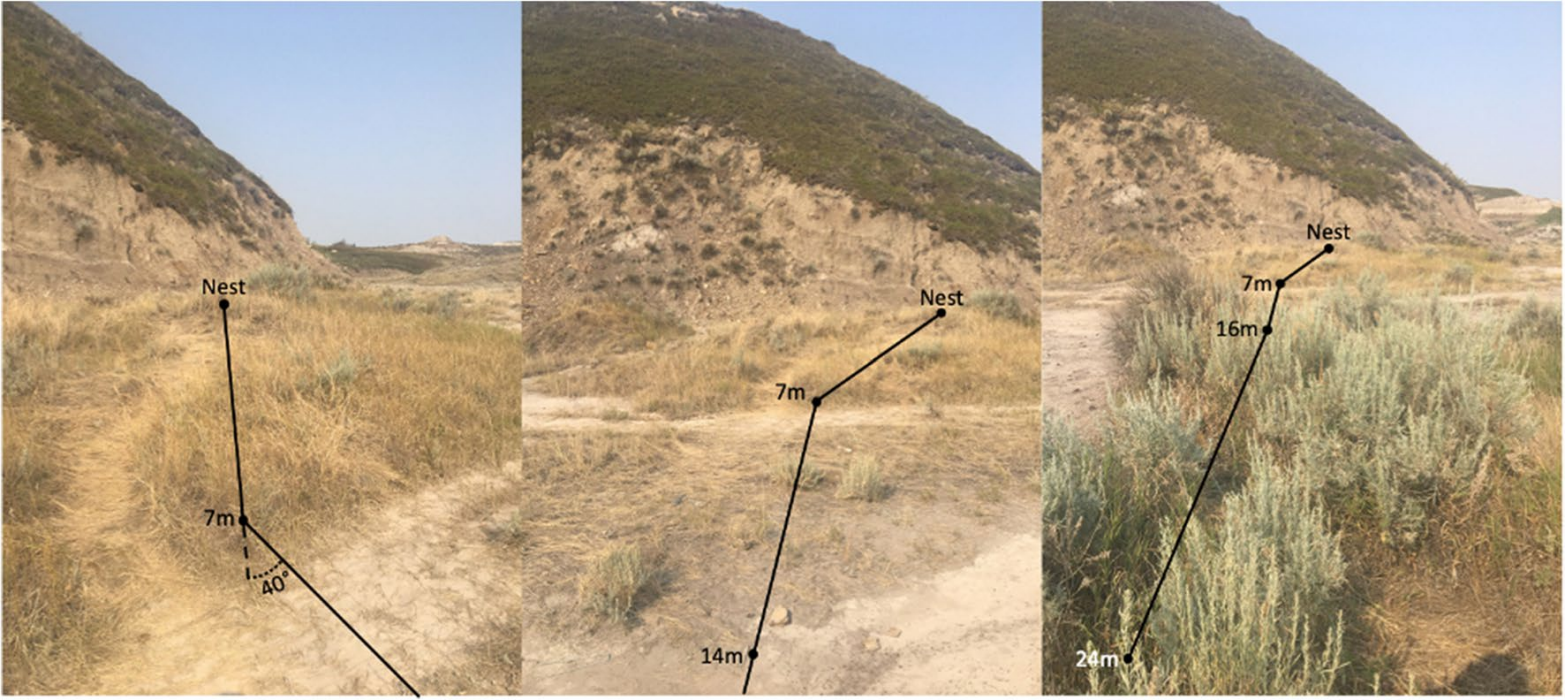
Photos of the *F.* *obscuripes* column with distinct segments from the nest to a group of bushes where foragers spread out to collect food at 24 m. This non-straight-line route contains a 40° counter-clockwise turn at 7 m from the nest, as well as a 7° clockwise turn at 16 m along the column. Photos exhibit the degree of visual cues available to navigating individuals and the changes in clutter over the route

This hybrid foraging structure, with individuals relying on a pheromone column before leaving the column to navigate solitarily during distinct periods of the journey, is not exclusive to *V.* *pergandei*. The western thatching ant (*Formica* *obscuripes*) also initially relies on a pheromone-marked column when leaving the nest, which shares some similarities to the column-and-fan structure of *V.* *pergandei*. Yet in *F.* *obscuripes*, the pheromone column extends out to stands of *Artemisia* bushes where foragers either climb into these bushes to farm Sternorrhyncha (collecting honeydew from aphids) or fan out along the surrounding ground and branches to hunt for other arthropods (Fig. 1). *F.* *obscuripes* is known to inhabit a variety of visually cluttered environments across its range (Glasier et al., 2014; Mackay & Mackay, 2002; Wheeler & Wheeler, 1986), suggesting it may rely on panoramic views to orient. Yet, little is known of the navigational abilities *of F. obscuripes*, and the navigational mechanisms they employ while travelling in either the presence or the absence of their pheromone.

Our initial aim in the current study was to characterize the navigational capabilities of *F.* *obscuripes*, a species inhabiting an array of habitats across a large geographic range throughout North America, yet has not been studied for its navigational behaviour (Wheeler & Wheeler, 1986). Specifically, we sought to discover if the mechanisms underlying navigation in the presence or absence of the pheromone cue, including orienting, path integration and backtracking, aligned with the only other studied fan-and-column foraging species, *Veromessor pergandei*. We began by conducting distant displacement tests to determine if heading behaviour was consistent with celestial compass-based path integration. Observations during these tests uncovered an interesting and previously undocumented behaviour of inbound foragers where foragers initially ‘retreat’ after release by first travelling in their outbound direction before altering their headings to celestial-compass-based inbound orientation, consistent with following a vector. We have classified this behaviour as retreating as it consists of abandoning active navigation to the current goal (nest). Retreating is often classified in outbound ants when foragers stop homing to a resource to return to the nest when cues change (i.e., when the celestial compass is altered; Freas et al., 2017b), however, the consistency between outbound and inbound retreating is the abandonment of the current goal and a return in the direction recently travelled. Typically, when outbound orientation is observed in inbound ants, it is associated with backtracking, a behaviour that occurs when foragers are close to the nest and have a near-zero path integrator state (Freas et al., 2019a; Plowes et al., 2019; Wystrach et al., 2013). Yet here the observed outbound headings occurred in foragers collected all along the column when much of their accumulated vector remained. Additionally, backtracking is considered a form of directed search, which persists as the forager’s search expands, inconsistent with this retreat behaviour (Müller & Wehner, 1994; Schultheiss et al., 2016; Wystrach et al., 2013). Here, outbound orientation was only temporary when foragers had most of their vector remaining, suggesting this behaviour is distinct from backtracking. We chose to characterize retreating behaviour, and the conditions under which it occurs, in a separate group of tests in addition to establishing the general navigational capabilities of this species.

## General methods

### Study site and species

Testing was conducted in July and September 2021 on a single *F.* *obscuripes* nest located at a field site within the Midland Provincial Park, Alberta, Canada (51°28′34′′N, 112°46′38′′W). *F.* *obscuripes* at this site forage within a visually cluttered environment, densely covered in grass tussocks interspersed with stands of *Artemisia* bushes and the occasional cactus (Fig. 1; for full panoramas see Online OSM Material (OSM) Fig. 1). *F.* *obscuripes* leave the nest in a pheromone-marked column that extends several meters before this trail ends and foragers spread out. Some foragers climb in the surrounding stand of bushes to farm Sternorrhyncha (aphids at this site), while others collect invertebrates in the surrounding brush (Glasier et al., 2014; Wheeler & Wheeler, 1986). Returning foragers re-enter the column and follow it back to the nest.

### Nest and foraging route

The *F.* *obscuripes* nest utilized in this research displayed a foraging column that was stable in its distance and direction over the course of testing over multiple months. Foragers would begin activity in the morning ~09:30 with foragers travelling along a set path 24 m to a line of bushes. There, foragers either spread out on the ground beneath these bushes collecting arthropods or climbed into one to tend Sternorrhyncha. All foragers were observed leaving and returning to the nest along the same route with morning activity ceasing ~11:30. No foraging activity was observed during mid-day, with another foraging bout along this stable route occurring in the late afternoon ~17:00 and ceasing before sunset, ~20:00.

**Fig. 2:**
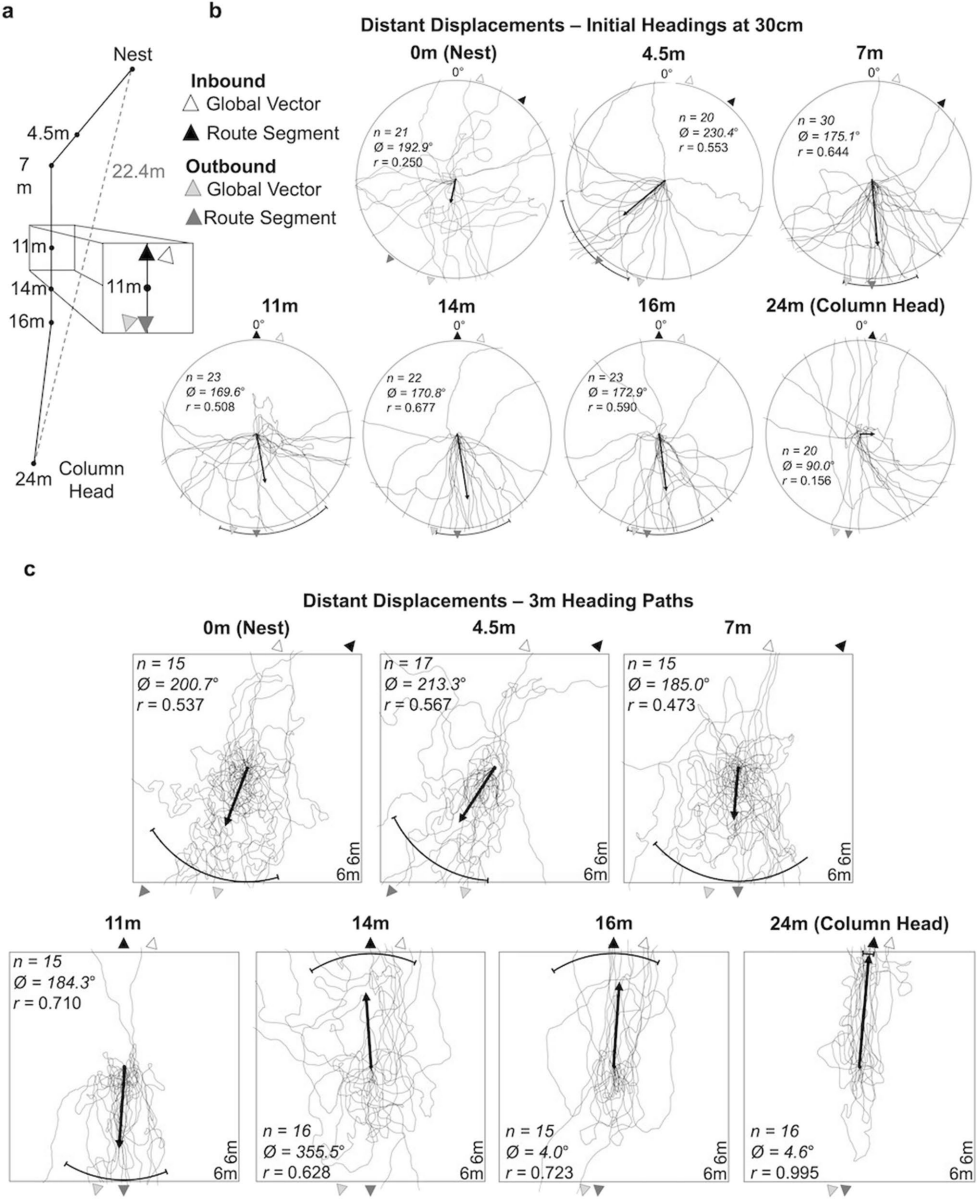
Diagram of the column and forager paths showing initial headings and 3-m paths at the distant site. **(a)** Column depicts the seven collection points for distant displacement testing. This column was stable across testing. **(b)** Each path crosses the circle at 30 cm from the release point and indicates the individual’s initial heading after release upon the reference circle board. **(c)** Forager paths at the distant site up to 3 m from release. Statistics refer to headings at 3 m; for 30-cm initial headings, see Online Supplemental Material Table 1. The white arrow depicts the compass direction of the individual’s inbound global vector compass direction from the column head to the nest entrance (14º), while the black arrow depicts the compass direction of the individual’s inbound route from its collection point. The light grey arrow shows the compass direction of the individual’s outbound global vector from the nest to the column head (194º), while the dark grey arrow depicts the compass direction of the outbound route from its collection point. The black bar represents the 95% confidence interval of headings in conditions where initial headings were directed, while arrows falling within that range indicate that headings are significantly directed in that predicted compass direction. The arrow emanating from each circle’s centre denotes the mean vector. *n*, number of individuals; Ø, mean vector; *r*, length of the mean vector

The trail foragers traversed was not straight, despite the lack of visible obstacles along the straight-line route to their foraging bushes (Fig. 1), though this area was depressed and filled with standing water for 2 days after heavy overnight rainfall, suggesting this area may not always be accessible to travel through. Foragers left the nest oriented 25° to right of the column’s end and this first column segment extended 7 m through thick brush before foragers made a 40° turn counter-clockwise with this second segment extending for 9.1 m across a relatively more open area (Fig. 1 and 2a). At this point, the column turned 7° clockwise and extended another 8 m, where the column ended (Fig. 2a). This end point represented a distance travelled along the foraging route of 24 m, yet the straight-line distance from the column’s end to the nest entrance was a shorter 22.4 m (Fig. 2a).

### General testing methods

Inbound foragers were collected and transported for testing in an opaque phial. For some tests, we recorded initial orientation by placing the ants on a 60 cm × 60 cm wooden board levelled on or above the ground using a spirit level. On the surface of this board was painted a 30-cm radius reference circle. Foragers were released onto the centre of this circle and their paths were recorded until they exited at 30 cm (initial headings) using an HD GoPro camera (30 fps and 3,840 × 2,160 pixels image size), which was positioned 1.0 m directly above the release point facing down. For other tests we created a grid of 1 m squares (using pegs and string) and each forager was released onto the ground within the grid. Forager paths were recorded by drawing the path on graph paper (during observation) until the forager left the grid area, and we used these paths to determine each forager’s initial heading direction (at 30 cm) and their ultimate heading, as they exited the grid. For both types of measurements, observed headings were initially compared to two directional predictions based on the inbound use of a celestial compass (see Fig. 2a for a diagram of these directions for the 11-m site): (1) the compass direction of the nest from collection, signifying the forager’s remaining global vector (*inbound global vector*) and (2) the compass direction of the individual’s inbound route segment at collection (*inbound route segment*). Because the observed initial headings in several conditions were directed, but the 95% confidence intervals (CIs) of headings were well outside these predicted directions, we added two additional predicted directions to the analysis: (3) the nest-entrance-to-column-head compass direction (*outbound global vector*) and (4) the outbound direction of the individual’s current route segment at collection (*outbound route segment*).

### Statistical analysis

Paths were first digitised using GraphClick by taking the forager’s position at 0.2-s increments (or 20 cm for the drawn graph paper conditions). Heading directions in each condition were analysed using the Oriana 4 Software Package for circular statistics (All Oriana Circular Statistics outputs are available at OSF https://doi.org/10.17605/OSF.IO/3CS6P). Conditions were first analysed to determine if they were statistically directed using Rayleigh Uniformity Tests for circular data (Fisher 1993, equations 4.17 and 4.18). If headings were considered directed via the Rayleigh Test (α = 0.05), we further analysed if the mean direction of observed headings was in a particular predicted direction using the 95% CI of headings; 95% CIs were calculated through the standard error of the mean heading direction based on the mean vector length (using Fisher 1993, equation 4.42). CIs for conditions that were not significantly directed via Rayleigh’s Tests were not reported as headings and were considered random. For this study, a predicted direction was considered to not statistically deviate from the mean heading if that direction fell within the 95% confidence intervals of observed headings. Comparisons of headings between conditions from release were conducted using Watson-Williams F-Tests (Fisher, 1993, p.126; Batschelet, 1981, p.95) while within-condition comparisons by distance were conducted using Moore’s Paired Tests (Zar, 1999; p. 647). When multiple comparisons were made, Bonferroni corrections (^*α*^*/*_*n*_) were used to determine significance level. Comparisons of the mirrored and unmirrored retreat conditions (Experiment 3) were also compared for heading variance differences using Var tests. Var tests consist of taking the absolute directional difference from each ant’s heading from condition’s mean vector direction, thus creating a metric for variance around this mean (for a full description, see Wystrach et al., 2014).

## Experiment 1: Distant displacements

### Methods

We began characterising the navigation of *F.* *obscuripes* by conducting two sets of distant displacement tests, in which inbound foragers were collected from multiple places along their foraging column and released at an unfamiliar distant location to establish orientation via the celestial compass. This allowed us to assess orientation behaviour when the surrounding panorama cues were unfamiliar as well as when foragers lacked their pheromone cue. In our first set of tests, we used the wooden board to record each forager’s initial headings at 30 cm (n = 159) and in our second set of tests, we used a 6-m × 6-m grid to record forager paths up to 3 m from their release (n = 109). For each set of conditions (initial orientation and 3-m path testing), inbound foragers were collected from one of seven locations along the column route; 0 m, 4.5 m, 7 m, 11 m, 14 m, 16 m, and 24 m (Fig. 2a), with 0-m foragers having completed almost the full return trip back to the nest (collected within 20 cm of the nest dome structure) and 24-m foragers being collected from the column head. Each forager was transferred to a distant testing site 120 m from the nest location. This distant testing site was chosen as it was flat, naturally clear of all local vegetation, and visually distinct from the nest site (OSM Fig. 1).

## Results

### Initial headings at 30 cm measured on the wooden board

Inbound foraging ants collected from the two ends of their 24-m long foraging column, just before entering the nest (0 m) or at the column’s head (24 m), and then released at the distant unfamiliar site, exhibited initial headings that were randomly distributed and not oriented in any direction, indicative of search behaviour (Fig. 2b; Rayleigh’s Test, p > 0.05). Conversely, inbound individuals collected at the five points along the column (4.5 m, 7 m, 11 m, 14 m, 16 m) exhibited directed initial headings at 30 cm (Rayleigh’s Test, p < 0.05; Fig. 2b; OSM Table 1), yet neither the *inbound global vector* nor the *inbound route segment* compass directions lay within the 95% CI of observed headings (OSM Table 1). When we added the two additional predicted outbound directions, the *outbound route segment* fell within the 95% CI of observed headings for all five conditions (Fig. 2b). Yet in three of these conditions (7 m, 11 m and 16 m) we could not differentiate between outbound directions as both the *outbound global vector* and *route segment compass* directions fell within the 95% CI of headings (Fig. 2b; OSM Table 1).

### Forager paths

When we measured paths up to 3 m, initial headings at 30 cm remained consistent with previous testing using the reference circle (Fig. 2bc), yet the observed outbound headings did not persist beyond this initial orientation in multiple conditions. Instead, individuals abandoned outbound orientation and significantly altered their headings beyond 30 cm. Inbound individuals collected at the nest (0 m) were initially not directed at 30 cm (Fig. 2c; Rayleigh’s Test, p > 0.05), yet by 3 m these same foragers became significantly directed (Rayleigh’s Test; *Z =* 4.32; *p =* 0.01) in the outbound compass directions (95% CI; Fig. 2c; OSM Table 1). After displacement from 4.5 m, 7 m and 11 m along the column, individuals were directed at both 30 cm and 3 m (Fig. 2c; Rayleigh’s Test, p < 0.05; OSM Table 1). In the 4.5-m displacement condition at 30 cm, only the *outbound route segment* and not the *outbound global vector* fell within the 95% CI. In the 4.5-m condition at 3 m, as well as at both 30 cm and 3 m in the 7-m and 11-m conditions, both the *outbound route segment* and the *outbound global vector* fell within this range (95% CI of observed headings; Fig. 2c; OSM Table 1). When individual headings in these three displacement conditions were compared between 30 cm and 3 m, mean headings did not significantly differ (Watson-Williams *F*-Test, p > 0.05).

In contrast, after distant displacement from 14 m or 16 m along the column, individuals were directed at both 30 cm and 3 m (Rayleigh’s Test, p < 0.05; OSM Table 1) but the direction of this orientation significantly changed between distances. Foragers in these two conditions were initially (at 30 cm) oriented in both outbound directions (95% CI; Fig. 2c; OSM Table 1), yet by 3 m individuals had significantly altered their mean headings (14 m: Watson-Williams *F*-Test, *F*_(1,30)_* =* 41.3, p < 0.001; 16 m: *F*_(1,28)_* =* 36.1, p < 0.001) and paths were now directed towards the inbound compass directions, though no determination could be made between orientation to the *inbound global vector* or the *inbound route segment* directions as both fell within the 95% CI of headings (Fig. 2c; OSM Table 1). Foragers collected from the column head at 24 m did not exhibit directed initial headings at 30 cm (Rayleigh’s Test, p > 0.05; OSM Table 1), but these same foragers were directed by 3 m from release (Rayleigh’s Test, *Z =*15.9, *p* < 0.001) and only the *inbound route direction* was within the 95% CI of headings while the *inbound global vector* lay outside this range (Fig. 2c; OSM Table 1).

## Discussion

The direction of initial orientation when released at distant sites depended upon the location along the column at which the inbound ants were collected. For inbound ants collected within the column, distant testing uncovered an ‘initial retreat’ behaviour, in which individuals released distantly either on the reference circle board or on the ground initially oriented in the outbound compass direction. Interestingly, this retreating behaviour did not occur when collected near the nest (0 m) or at the column head (24 m). In both of those cases, initial orientation was random. Analysis of forager paths over longer distances revealed that individuals in all groups recovered from their initial retreat or search behaviour and showed significant orientation by 3 m. However, the direction of orientation again depended on collection location. For ants collected within 11 m of the nest, orientation was in the outbound direction, reflecting backtracking behaviour. However, ants collected at 14, 16 or 24 m from the nest (with over 50% of their column distance remaining) oriented in the inbound direction. This suggests that backtracking in this species relies, at least in part, upon the remaining portion of their accumulated global vector.

## Experiment 2: Characterizing retreating behaviour

### Methods

#### On-column tests

To further explore the retreating behaviour discovered in Experiment 1, we tested for retreating behaviours while the individual remained in the pheromone column, after a disruption along the inbound route. This would test if the observed retreating behaviour is connected to navigational uncertainty and thus is influenced by the lack of familiar cues at the distant site. We erected a 4 × 4 grid of 50 cm squares (2 m × 2 m) around a site along the column 14 m from the nest. We collected inbound foragers from two points along the column, at 7 m (n = 16) and 14 m (n = 16), because behaviours on the distant displacement tests pointed to distinct strategies at different distances: Individuals collected at 7 m initially retreated and then continued to travel in the outbound direction (backtracking) while individuals collected at 14 m initially retreated before turning and travelling in an inbound direction, consistent with following a path integrator. Foragers collected from these two points along the column were released back onto the foraging column at the 14-m site (two researchers were present during testing to maintain continuous visual contact with the focal animal). Individual paths were recorded until they left the grid, with heading directions recorded at 30 cm and 1 m. We tested the measures against all four predicted directions: (1) *Inbound remaining vector* from its collection site and (2) the *Inbound* *current route* direction at the 14-m release site. We also tested two outbound directions representing the outbound directions, (3) *Outbound remaining vector* and (4) *Outbound current route.*

As foragers still exhibited an initial retreating behaviour despite being displaced back into the column, we theorised this behaviour was an aversive behavioural response to collection and release. As a similar, though undirected, bolting behaviour occurs is bull ants (In *Myrmecia midas*, see Freas et al., 2017a), we developed a follow-up set of displacement conditions designed to reduce this behaviour. We restricted the forager’s initial horizontal movement by forcing them to climb down a small distance before choosing a heading (inversely mimicking the climb up methodology used in *Myrmecia midas*; Freas et al., 2017a; Freas & Cheng 2019). At the 14-m release site we sunk a 15-cm wooden peg into the ground with 10 cm of the length remaining above ground located in the centre of the foraging column’s width. Inbound individuals (n = 16 each site) were again collected (from either 7 m or 14 m) and displaced to the 14-m site. In these conditions, foragers were released onto the top of the wooden peg, forcing them to descend 10 cm to the ground before choosing a heading. Individual paths were recorded until they left the grid with headings recorded at 30 cm and 1 m. We tested this set of conditions against the same four predicted directions.

### Local lateral displacements

Local displacements around the column were expanded by extending the 2 m × 2 m grid to a 4 m × 8 m grid. This was accomplished by extending 3 m to the right of the edge of the column, 7 m inbound and 1 m outbound from the 14-m release point (Fig. 4). Identical to the wooden peg at the 14-m on-route release point, a second peg was placed 2 m lateral to the right of this site, off the pheromone column (Fig. 4). Inbound foragers were collected from two points along the column, 7 m, and 0 m (where we observed backtracking when the surrounding panorama was unfamiliar) and then transferred locally to the 14-m release point. The first group of individuals (n = 12 each condition) were placed onto the peg that was on the column, while a second group (n = 12 and 16, respectively) was released onto the peg location 2 m laterally to the right of the column. Paths were recorded by observing and recording the path on graph paper until the foragers exited the grid. Heading directions were recorded at 30 cm, 1 m and the exit direction.

### Above the column

Believing that we had sufficiently eliminated the expression of retreating behaviour after displacement by forcing displaced ants to climb down before setting a heading, we conducted a set of tests on the wooden board above the column. This testing removed the ants from the pheromone trail but gave them access to the familiar surrounding visual panorama, allowing us to test if foragers relied on this information when homing. The wooden board was fitted with a 10-cm wooden peg embedded at its centre and was placed with its edge at 14 m along the foraging column and raised 10 cm above the ground using four flat-top stakes (Fig. 5). Inbound individuals were collected from two points along the foraging column, either at 14 m (n = 29) or just before reaching the nest (zero vector; n = 23). Each ant was transported to the board where they were individually released at the top of the wooden peg and forced to climb down to the board’s surface before choosing a heading direction. Individuals’ paths were recorded from the centre point to the board’s edge (30 cm).

As we again observed retreating behaviour in 14-m individuals and random headings in zero-vector individuals (consistent with distant testing), despite the climb-down mitigation method, it appeared that either foragers were unable to orient above the column using familiar views, or the board was contributing to the observed retreating. We hypothesised that the unfamiliar substrate of the board surface may elicit the observed retreating behaviours. We therefore conducted a second set of above column conditions, identical to the previous conditions except that we added a thin layer (~2 cm) of soil (collected from 30 m away from both the nest and column) over the surface of the board. Foragers from the two collection sites (n = 20 each) were displaced to the board and their headings were recorded using the overhead camera. We assessed the directionality of these conditions and we compared the mean vector directions between substrate conditions.

### Distant sun mirroring

In a final round of retreating focused testing*,* we sought to determine if the celestial compass influenced the direction of retreating behaviour. Here, we conducted a set of conditions setting these celestial cues in conflict by mirroring the sun’s azimuthal position (the sky’s polarisation pattern remains unaltered). We again collected individuals from 14 m along the column and released them at the distant site. Given our previous testing on retreating, we sought to maximise foragers’ propensity to retreat by releasing them directly onto the surface of the wooden testing board. In the unmirrored control (n = 22), all celestial cues remained unaltered. In the sun-mirrored condition (n = 21), we blocked the sun’s true azimuthal position in the sky and used a 30 cm x 30 cm mirror to replicate the sun in 180° conflict with the sun’s true position. Forager paths were recorded until they crossed the 30-cm reference circle.

## Results

### On and around the column

#### Retreating on the column

**Fig. 3:**
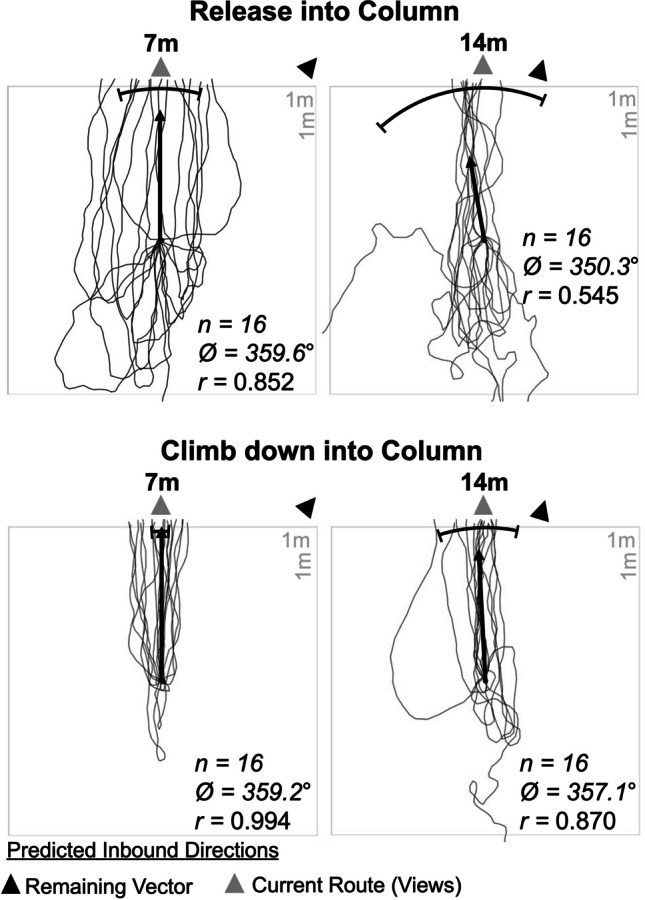
Forager paths when released back into the column either by releasing them directly into the column or forcing them to climb down into the column from a 10-cm wooden peg. Forager paths are shown until they exited the 2 m × 2 m grid, 1 m from release. Statistics refers to foragers’ headings at 1 m; for 30-cm initial headings see Online Supplemental Material Table 2. The grey arrow depicts the inbound direction of the column at the release point (0º), while the black arrow indicates the compass direction for the forager’s remaining path integration derived vector. The black bar represents the 95% confidence interval of headings, while the arrows falling within that range indicate that headings are significantly directed in that predicted direction. The arrow emanating from each release point denotes the mean vector. *n*, number of individuals; Ø, mean vector; *r*, length of the mean vector

Inbound ants collected from 7 m or 14 m, then released at 14 m directly back onto the foraging column exhibited directed initial headings at 30 cm (Rayleigh’s Test, *p* < 0.05), and these headings were in the outbound directions (95% CI of headings; OSM Table 2), though no determination could be made regarding if foragers were following their *Outbound remaining vector* or the *Outbound current route* direction (Fig. 3). By 1 m, individual headings were still directed (Rayleigh’s Test, *p* < 0.05; OSM Table 2) but headings had changed significantly in direction from initial headings at 30 cm (7 m; Moore’s Paired Test *R*_(16)_* =* 1.955, p < 0.001; 14 m; *R*_(16)_* =* 1.592, p < 0.001). Foragers collected from 7 m were directed only in the direction of the *Inbound current route* segment (0º), while these foragers showed no evidence of orientation to their *Inbound remaining vector* at 40º (95% CI; Fig. 3; OSM Table 2). In 14-m foragers, the *Inbound remaining vector* and *Inbound current route* segment directions were quite close directionally (17.4º and 0º respectively) and both directions fell within the 95% CI of headings (Fig. 3; OSM Table 2).

Inbound ants released on top of the wooden flat-top peg and forced to climb down into the column did not exhibit the same retreating behaviour, with 7-m foragers being directed (Rayleigh’s Test, *p* < 0.05; Fig. 3) in the direction of only the *Inbound column route* at both 30 cm and 1 m (95% CI; OSM Table 2). Foragers collected from 14 m did not exhibit directed initial headings (Fig. 3; Rayleigh’s Test, *p* = 0.065), suggesting that while this release method may reduce retreating, it did not eliminate the behaviour (Fig. 3; four of 16 foragers in this condition still initially retreated). By 1 m these foragers’ headings were directed (Rayleigh’s Test, *Z =* 12.1*, p =* 0.001) and only in the direction of the *Inbound current route* and not the *Inbound remaining vector* or either outbound direction (95% CI; OSM Table 2).

#### Local lateral displacements

**Fig. 4:**
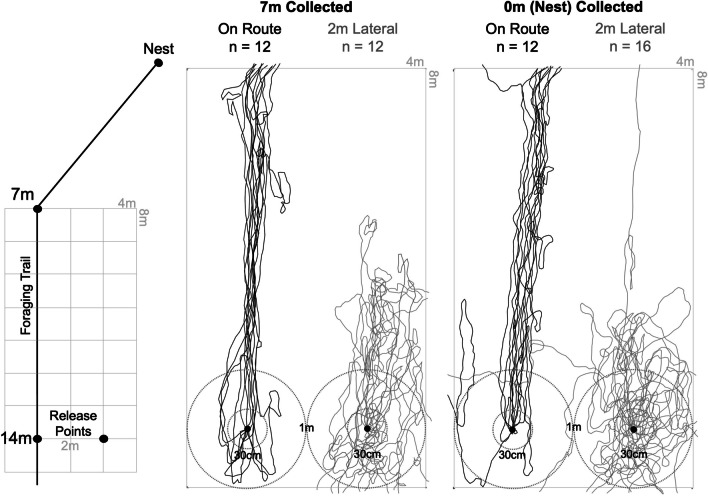
Diagram of the grid layout in relation to the column and 2-m lateral displacement sites and forager paths in the four conditions. Inbound foragers were collected from the column at either 7 m or just before entering the nest and released back at 14 m either on the column (black paths) or 2-m lateral off the column (grey paths). To reduce retreating behaviour, each forager was released onto the top of a 10-cm high peg and had to climb down to the ground prior to horizontal movement. Headings were recorded at three distances (30 cm, 1 m, and grid exit). Distances of 30 cm and 1 m from the release point are marked by the dotted circles

Foragers collected at 7 m and released back to the 14-m site were not initially oriented, with a number of individuals initially retreating at 30 cm (Rayleigh’s Test, *p* > 0.05; Fig. 4; OSM Table 2), yet forager headings at 1 m were directed (Rayleigh’s Test, *p* < 0.05) in only the inbound *current route direction* (95% CI; OSM Table 2).

When analysing if this directed behaviour persisted until grid exit, we found that while foragers remained directed, paths were slightly biased to the right and thus no direction was within the 95% CI of headings (2°–4°) with the *inbound current route* direction being just outside this range at 0° (Fig. 4). As foragers appeared to turn clockwise within the last 50 cm of the grid, corresponding to the point the column turns 40° towards the nest at 7 m (Fig. 4, black paths as they exit the top of the grid), we suspect that we are picking up the initial part of this turn, with foragers altering their orientation along the two route segments, thus leading to the slight rightward bias in headings. Finally, there was no significant difference in mean heading direction between 1 m and exit distances (Moore’s Paired Test *R*_(12)_* =* 0.873, p > 0.05).

When 7-m individuals were displaced 2 m laterally, they were initially not oriented at 30 cm (Rayleigh’s Test, *p* > 0.05), but were oriented by 1 m (Rayleigh’s Test, *p* < 0.05; OSM Table 2) and were directed in both inbound directions (95% CI). Mean heading direction significantly shifted (Moore’s Paired Test *R*_(12)_* =* 1.593, p < 0.001) by the time they exited the grid, with these headings being directed (Rayleigh’s Test, *p* < 0.05) in the direction of only the *outbound current route* at 180° (95% CI; Fig. 4; OSM Table 2).

Individuals collected at 0 m and released back within the column to the 14-m site were initially oriented at 30 cm, 1 m and at the grid exit distances (Rayleigh’s Test, *p* < 0.05) and directed only in the *Inbound current route* direction, suggesting they followed the column back towards the bend at 7 m to the nest (95% CI; Fig. 4; OSM Table 2). When 0-m foragers were released at the off-pheromone site 2 m lateral from the 14-m site on the column, headings were directed at all distances (Rayleigh’s Test, *p* < 0.05), yet headings were at first directed, at 30 cm and 1 m, to both inbound directions (95% CI; Fig. 4; OSM Table 2), and mean headings did not differ at these two distances (Moore’s Paired Test, *R*_(16)_* =* 0.631, p > 0.05). By exit, headings significantly shifted (Moore’s Paired Test, *R*_(16)_* =* 1.476, p < 0.005) to become directed to only the *outbound current route* direction by the time foragers exited the grid (95% CI; OSM Table 2). Headings of both 7-m and 0-m foragers suggest that while individuals could initially orient correctly to the inbound route when displaced locally off the pheromone, there was insufficient information even 2 m off the route for successful continued orientation and these foragers ultimately backtracked.

#### Above the column

**Fig. 5:**
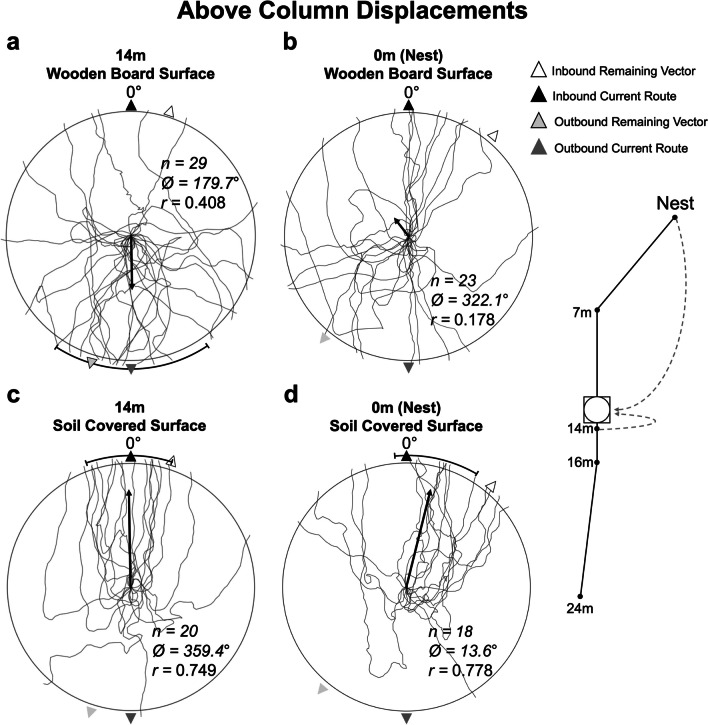
Diagram of the procedure for collecting foragers and displacing them to the reference circle board located 10 cm above the 14-m on-column site. Forager paths showing initial headings when displaced 10 cm above the column on a board. Each path crosses the circle at 30 cm from the release point and indicates the individual’s initial heading after release upon the reference circle board. The white arrow depicts the compass direction of the individual’s remaining inbound vector, while the black arrow depicts the compass direction of the inbound route at the 14-m site. The light grey arrow shows the outbound compass direction of the individual’s remaining vector, while the dark grey arrow depicts the outbound direction of the route at the 14-m site. The black bar represents the 95% confidence interval of headings in conditions where initial headings were directed, while arrows falling within that range indicate headings are significantly directed in that predicted compass direction. The arrow emanating from each circle’s centre denotes the mean vector. *n*, number of individuals; Ø, mean vector; *r*, length of the mean vector

In our conditions with a wooden board placed above the column, when foragers were collected from 14 m and displaced above the column, these forager headings were directed (Rayleigh’s Test, *Z =* 4.8*, p =* 0.007), yet only the outbound directions were within the 95% CI of headings (Fig. 5a; OSM Table 2). Foragers collected with no remaining path integrator and displaced above the column at 14 m were not directed (Rayleigh’s Test, *Z =* 0.726*, p =* 0.489; Fig. 5b). This pattern of results is consistent with the retreating behaviour we observed at the distant site.

When these above-column conditions were repeated with a soil-covered board, displaced foragers exhibited significantly different initial headings. Individuals collected at 14 m were still directed (Rayleigh’s Test, *Z =* 11.207*, p < 0.001*), but now in the inbound direction, with both the remaining vector and current route directions within the 95% of CI of headings (Fig. 5c; OSM Table 2). Additionally, mean initial heading directions between the wooden and soil-covered 14-m conditions were significantly different (Watson-Williams *F*-Test, *F*_(1,47)_* =* 64.557, p < 0.001). A similar change occurred in inbound foragers collected at the nest with no directional path integrator information: foragers released on the soil covered board were directed (Rayleigh’s Test, *Z =* 10.906*, p <* 0.001), yet only the inbound current route direction was within the 95% of CI with their final inbound segment (40º) falling outside this range (Fig. 5d; OSM Table 2). Finally, there was no significant difference in heading direction between the two conditions (collected at the nest or at 14 m) when tested with soil covering the board (Watson-Williams *F*-Test, *F*_(1,36)_* =* 1.021, p = 0.319)

### Distant sun mirroring

**Fig. 6:**
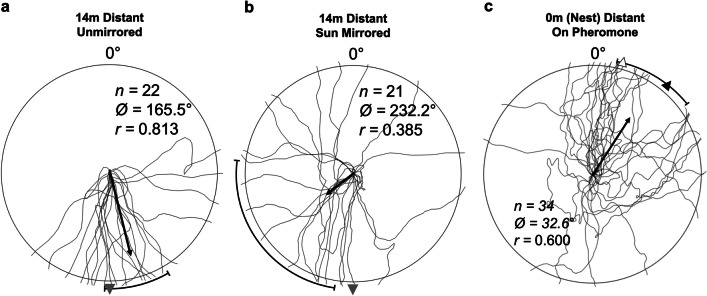
Forager paths indicating initial headings at 30 cm for inbound individuals in the sun-mirroring conditions (**a** – control; **b** – sun mirrored) and the (**c**) distant pheromone condition. The grey arrow in the mirror conditions shows the compass direction of the outbound route segment at the 14-m site. In the distant on-pheromone condition, the white arrow denotes the compass direction of the forager’s full vector memory while the black arrow signifies the compass direction of the forager’s last vector segment (from 7 m to 0 m). The black bar represents the 95% confidence interval of headings in conditions where initial headings were directed, while arrows falling within that range indicates headings are significantly directed in that predicted compass direction. The arrow emanating from each circle’s centre denotes the mean vector. *n*, number of individuals; Ø, mean vector; *r*, length of the mean vector

In our distant displacement control, released foragers reacted identically to our original initial heading conditions with distant displacements. Forager headings were directed (Rayleigh’s Test; Z = 14.543; p < 0.001; Fig. 6a) and the outbound route segment at 180° was within the 95% CI of headings (*θ* = 165.6 ± 15.2°). In sun-mirrored foragers, headings were only very weakly directed (Rayleigh’s Test; Z = 2.30; p = 0.043; Fig. 6b). With such weak orientation, 95% Cis become very large and unreliable, yet the outbound route segment was still outside the 95% CI of headings (*θ* = 232.2 ± 43.3°). Mean heading directions between mirrored and control conditions were significantly different (Watson-Williams *F*-Test, *F*_(1,42)_* =* 10.79, p = 0.002) and the amount of directional variance was also significantly higher in the sun-mirrored condition compared to the control (Var test; W = 71.0; p < 0.001), consistent with increased uncertainty in heading direction in sun-mirrored ants. These differences suggest that the sun’s position partially mediates the retreating response. Importantly, the incomplete shift in paths is consistent with the fact that the celestial compass comprises multiple celestial cues, placing the sun’s position in conflict with other cues such as the sky’s polarisation pattern, thus not fully mirroring the complete sky compass.

## Discussion

Our retreating results suggest the behaviour contains four predictable aspects. (1) Retreating occurred after collection from within the column but not the nest or column head. (2) Retreating is distinct from backtracking behaviour. (3) Retreating is not elicited via navigational uncertainty. (4) Retreat was in the direction of current route segment and not a global vector direction. Combined, the results point to retreat as being a goalless directed orientation behaviour elicited by aversive events and informed by the forager’s current vector state and guided by the celestial compass. Furthermore, *F.* *obscuripes* foragers show evidence both of vector maintenance and panorama use while navigating both on and off the pheromone cue. Importantly, the pheromone’s presence appears critical to continued orientation to these other cues with laterally displaced foragers ultimately abandoning inbound homing when not on the pheromone trail.

## Experiment 3: Testing the role of the pheromone in backtracking

### Methods

To assess if the presence of the trail pheromone plays a role in backtracking behaviour when the panorama is unfamiliar, we conducted a final condition at the distant site incorporating the measures to reduce retreating. A 60 cm × 60 cm wooden board was covered in a layer of soil and placed within the long-term foraging column (at the 14-m site) prior to the onset of morning foraging. The foraging column was allowed to form over the board and outbound foragers were observed to deposit pheromone along this area. The board was allowed to collect pheromone cues as the column formed over the board. We waited 1 h after column formation to confirm that the column had formed fully with no retreating or meandering observed on the board’s surface before transferring the board to the distant site. Here, the board was placed on the ground in the compass orientation of its position on the column and a 10-cm peg was placed in the centre. Inbound foragers were collected (n = 34) just as they reached the nest, transported to the distant site and then released at the top of the 10-cm wooden peg. Each forager was recorded as it travelled down onto the board and until it left the board’s surface at 30 cm (initial heading) using an overhead camera identical to previous initial-heading conditions. Between each forager the orientation of the board was rotated 180° to control for any potential directional information of the pheromone cue.

## Results

Inbound foragers collected just before reaching the nest, with a path integrator that would be directionally uninformative, and released distantly back on a pheromone trail exhibited initial headings that were directed (Rayleigh Test, *Z =* 12.23, *p* < 0.001; Fig. 6c) in the inbound route compass directions, though no determination could be made if foragers were oriented to the compass direction of their complete global vector (14°) or the last inbound segment (40°) as both directions fell within the 95% CI of headings (*θ* = 32.6 ± 20.3°). There was no difference between foragers tested with the pheromone aligned with the inbound compass direction and with the pheromone rotated 180° (Watson-Williams *F*-Test, *F*_(1,32)_* =* 0.002, p = 0.968).

## Discussion

Backtracking behaviour in ants collected just before entering the nest and tested distantly was substantially eliminated when the pheromone cue was present (compare Figs. 2b, 3, 4, 5 and 6c), and instead the ants oriented in the inbound direction. The elimination of backtracking did not depend on the directionality of the pheromone trail because rotation of the board containing the pheromone trail had no effect. These results (along with the findings from Experiment 1) indicate that *F.* *obscuripes* exhibits backtracking based on a combination of the factors previously observed in other ant species. Backtracking was observed when at an unfamiliar location with less than 50% homeward route remaining, indicating that recent exposure to the nest’s panorama was not a critical trigger as reported in *M.* *bagoti* (Wystrach et al., 2013; OSM Fig. 1). However, the results of testing on the board above the column, together with the distant pheromone testing results, suggest that *F.* *obscuripes* can rely either on the familiarity of the current panorama or the presence of the pheromone when deciding to not backtrack. Backtracking occurs only in the absence of both cues, which is distinct from both solitarily foraging ants and from trail following *V.* *pergandei* (Wystrach et al., 2013; Freas et al., 2019a).

## General discussion

### Summary

Our findings can be separated into two themes. First are those concerning the general navigational abilities of *Formica* *obscuripes* during foraging and how it compares to other ant species. Second is a discussion of the novel directed retreating behaviour and its underlying mechanisms as well as how it fits within this species’ foraging ecology. On the navigation theme, distant displacement headings suggest that the ants maintain a path-integration-derived vector while inbound on the pheromone column, whereas displacements on, above and near the column show a reliance on familiar panoramas for guidance. Additionally, there is clear evidence of backtracking behaviour in this species distinct from their initial retreating behaviour. Backtracking appears to be mediated by a combination of visual cues and the pheromone, ultimately showing a combination of mechanisms observed in other ant species where this behaviour has been characterised.

### Navigational strategies

#### Path integration

The maintenance of a path-integration-derived vector, reliant on the celestial compass, is well modelled in ants (Collett & Collett, 2000; Wehner, 2020; Wehner et al., 1996). Additionally, the presence of a path integrator is known to run both in many individually foraging species as well as in socially foraging species while on a pheromone trail (Collett & Collett, 2000; Freas et al., 2019b; Heinze et al., 2018; Wehner & Srinivasan, 2003). In socially foraging species, this vector is thought to provide directional information on the trail as non-bifurcating pheromone trails lack inherent polarity (Czaczkes et al., 2015; Freas et al., 2021; Minoura et al., 2016).

Much like other ant species, *F.* *obscuripes* foragers show evidence of vector maintenance, using both directional compass cues as well as distance estimates to orient when placed at a distant unfamiliar site, both aspects of a continuously operating path integrator. Forager paths showed clear orientation to the compass direction associated with their inbound route (after their initial retreat) when they had > 50% of their column distance remaining. In contrast, once foragers had completed 50% of their inbound column distance, they began to orient in the opposite compass direction, consistent with backtracking behaviour, which is known to rely on the celestial compass and odometer cues (Freas et al., 2019a; Plowes et al., 2019; Wystrach et al., 2013). Additionally, when inbound foragers are collected at the nest, with their path integrator directionally uninformative, and displaced distantly to a pheromone-marked board, they continued to orient to their inbound vector direction rather than backtrack. This inbound direction was observed even when the pheromone board was rotated by 180°, thereby ruling out control of the inbound behaviour by directional cues from the pheromone trail. These results indicate two aspects of the relationship between the pheromone cue and the forager’s path integrator, which are identical to interactions observed in *V.* *pergandei* (Freas et al., 2021). First, it is further evidence of a lack of inherent pheromone directionality as there was no difference in headings with the pheromone in agreement or conflict with the vector compass direction with the lack of inherent polarity in straight pheromone trails being established in multiple ant species (Czaczkes et al., 2015; Freas et al., 2021; Minoura et al., 2016). Instead, the presence or absence of the pheromone, along with the proportion of the vector completed, dictates whether foragers continue to orient to their homeward vector or begin backtracking. Secondly, like *V.* *pergandei* (Freas et al., 2021)*,* foragers continued to orient to the inbound vector compass direction despite having a directionally uninformative vector state. Given the inherent accumulation of error in the path integration system, when the remaining vector state is near zero, yet the forager is still in contact with pheromone, it is likely advantageous to continue orienting in the inbound vector direction, rather than engage in search or backtrack as the pheromone’s presence indicates the nest has not yet been reached. However, the memory dynamics, such as whether foragers rely on a reloading of a long-term memory during this testing, remain unknown. The results are ultimately indicative that the pheromone acts as a context cue in how foragers choose their headings based on their path integrator, just as observed in *V.* *pergandei* (Freas et al., 2021).

#### Panorama cues

Forager headings in multiple local displacement tests show evidence of the use of familiar panoramic views to orient while near known locations. In conditions where foragers were released back into the column at 14 m, individuals oriented in the direction of the route’s views even when it conflicted with their remaining vector state (7-m condition), suggesting this vector is being overridden by view alignment. When we collected the full paths of foragers released back into the pheromone column from 14 m, foragers correctly follow this column even in the absence of a corresponding vector cues. As the path integrator is continuously running, foragers collected at the nest (0 m)

were likely accumulating a directionally conflicting vector in opposition with these views, yet still showed little hesitation to follow the inbound column. Just as with the path integration system, the presence of the pheromone appears to act as a context cue in relation to view alignment strategies. Though importantly, these on-pheromone paths could be accomplished through initially recognising the polarity of the pheromone trail via views or a vector (depending on condition) and then using the pheromone alone to navigate.

In the above-column and 2-m lateral displacements, which were both off the pheromone, headings were initially oriented to the correct inbound view direction (up to 1 m). However, 2-m lateral displacement paths suggest that foragers ultimately could not follow these views home or to the column in the absence of the pheromone and instead they appeared to quickly abandon this orientation in favour of backtracking.

Results indicate that, like other trail following ants (Czaczkes et al., 2015; Freas et al., 2021; Minoura et al., 2016), in *F.* *obscuripes* the pheromone cue alone contains no directional information, yet its presence informs how foragers use their directionally based navigational systems. Specifically, the pheromone’s presence acts to decrease uncertainty, consequently increasing the weighting given to navigational systems (path integration or view memory) over search behaviours, including backtracking. In the absence of the pheromone cue, weighting of these same navigational systems is suppressed, leading foragers to engage in search despite familiar views or a remaining path integrator. In this way, the pheromone acts as a verification or reassurance cue confirming with its presence that the foragers are travelling in the correct direction and should continue to follow these cues. The pheromone’s function to reassure foragers they are on the correct path has been documented in other ant species (Czaczkes et al., 2011; Freas et al., 2021; Wetterer et al., 1992) suggesting it may play a similar function in all trail following ants.

#### Backtracking

Navigational strategies (path integration and view memories) typically fail to return individuals to the exact location of the nest. Thus, navigating ants employ a number of back-up mechanisms during this final stage of their journey to pinpoint their goal. These include systematic search behaviour (Schultheiss & Cheng, 2011; Schultheiss et al., 2015; Wehner & Srinivasan, 1981), which occurs both in familiar and unfamiliar locations, and a behaviour called backtracking. Backtracking is thought to be widespread in navigating Hymenoptera (Collett & Collett, 2009; Collett et al., 1993; Freas et al., 2019a), yet its mechanisms have only been described in two ant species, a single solitarily foraging species, *Melophorus bagoti* (Wystrach et al., 2013) and more recently a single fan-and-column foraging species, *V.* *pergandei* (Freas et al., 2019a; Plowes et al., 2019). This behaviour sees inbound foragers, displaced just before entering the nest to an unfamiliar location, orienting in the opposite (i.e., outbound) direction they were previously heading before displacement, instead of a random direction indicating systematic search. Backtracking behaviours are theorised to aid foragers who have overshot the nest and are now in an unfamiliar area, leading them to return back along their route to reach more familiar locations. This behaviour only occurred in *M.* *bagoti* foragers under the combination of three criteria: (1) individuals had a path integrator state near zero, (2) individuals were presented an unfamiliar terrestrial panorama, and (3) individuals had recently been exposed to the nest panorama. These triggering conditions are not ubiquitous across ant species, with *V.* *pergandei* using the presence/absence of the trail pheromone instead of panorama familiarity to trigger backtracking. Additionally, *V.* *pergandei* did not require a path integrator state at or near zero, using instead the proportion of the homeward route (~75%) they ran off, which triggers backtracking, meaning the nest panorama is not a universal trigger (Freas et al., 2019a).

Here, *F*. *obscuripes* show a combination of the criteria observed in other ant species. First foragers were observed to backtrack when displaced to a distant, unfamiliar location when they had completed between 54% and 100% of their homeward route, with foragers collected even 11 m from the nest exhibiting backtracking. This is almost double the remaining path integrator proportion (46% vs. 25%) observed in *V.* *pergandei* and well beyond the nest’s panorama critical for backtracking in *M.* *bagoti *(OSM Fig. 1)*.* Second, foragers appear to use familiar panorama views when deciding to backtrack as they do not exhibit backtracking when exposed to the familiar panorama just above the column with no accompanying pheromone cues. Foragers can also use the presence of the pheromone when deciding to backtrack as zero vector foragers displaced distantly to an unfamiliar panorama onto the pheromone do not backtrack and instead orient to their inbound vector. This suggests that backtracking in *F*. *obscuripes* meets backtracking criteria of both previous species. Like *V.* *pergandei,* they clearly use the proportion of their column they have run off and not the nest panorama to initiate backtracking. Yet unlike *V. pergandei,* in this species backtracking only occurs in the absence of both the pheromone and a familiar panorama.

One complication of this description of backtracking is our results during 2-m lateral displacements. Here, foragers with little or no column distance remaining were presented a panorama that only differed slightly from their route panorama at 14 m (OSM Fig. 1), with foragers showing an ability to orient to these inbound views for at least 1 m. Yet by the time these foragers exited the grid, both groups of foragers (7 m and 0 m) were oriented in outbound directions consistent with backtracking. As noted above, this suggests that the presence of the pheromone is likely necessary as a verifier for continued view-based homing. In the pheromone’s absence, foragers will still ultimately choose to backtrack to attempt re-enter the pheromone column rather than face the challenge of travelling to the nest off the pheromone trail.

### Retreating behaviour

*Formica obscuripes* appears to be more sensitive to disturbance than many other species which typically don't show retreating in response to either collection or testing on an unfamiliar substrate. The observed initial retreating behaviour in *F*. *obscuripes* exhibited four predictable characteristics. First, retreating occurred after collection at all points within the column length but not at its two ends (at 0 m and 24 m), where forager headings were initially random. Second, this retreat was distinct from backtracking behaviour. Third, foragers exhibited retreating behaviour both when the available navigational cues upon release were familiar as well as when individuals were released distantly to an unknown location, suggesting the behaviour is not elicited via navigational uncertainty. Finally, while directional differences between the global vector and the current route segment were often small, retreating foragers in multiple conditions only significantly oriented to their current route segment when retreating and not a global vector direction.

Together the findings point to a celestial compass based, non-goal-directed orientation response elicited by the experimental manipulations and informed by the forager’s current vector state. This suggests retreating behaviour is a response to aversive events meant to facilitate escape while remaining on the pheromone marked column. This would align with retreating only being observed when their vector state indicates they were positioned within the column and not its ends. If a forager experiences an aversive event with a vector state indicating it is at the column head, retreating in an outbound direction where no pheromone exists would push them to travel off the column, increasing the chance they become lost. This may also explain the lack of retreating with a near zero vector state (while these same foragers still exhibited backtracking). With the safety of the nest so close, retreating away from the nest may also be disadvantageous for an anti-predatory response, leading to longer periods outside of the nest. This is in clear contrast to backtracking behaviour where the absence of the pheromone with a near-zero vector state means the forager may have passed the nest and should search the in opposite compass direction.

An important aspect of the observed retreating behaviour is that it occurs regardless of the level of navigational uncertainty present when orienting. Retreating occurs at the distant testing site or when displaced back into their foraging route, suggesting the degree of familiar cues present (view memories and pheromone presence) do not influence this behaviour. Additionally, displacements in route-following ants to other parts of their foraging route, thus placing the expect views out of order, have been shown to influence navigational uncertainty and increase hesitation behaviours (Schwarz et al., 2020). Yet here we observed no difference in retreating behaviour based on whether foragers were released to a familiar but unexpected view sequence of the inbound route (7-m or 14-m foragers released back on column at 14 m), suggesting that this uncertainty also had little or no influence on retreating. Instead, retreating appears to be triggered by the collection-and-release procedure, with foragers released directly onto the ground or board exhibiting the behaviour. Once we implemented the procedure to force foragers to descend 10 cm before choosing a heading, delaying the period between release and foragers choosing a heading, we observed a significant decrease in initial retreating behaviour.

Additionally, while navigational cue presence was shown not to influence this behaviour, the familiarity of the ground’s substrate did appear to affect retreating. When we released foragers just above their foraging column on a wooden board, they retreated even in the presence of familiar panorama cues. Yet, we were able to extinguish this retreating behaviour by spreading a familiar soil substrate (from a location off the pheromone column) over the board’s surface. These results suggest that the unfamiliar wooden substrate caused a neophobic response from the ants, with continuous contact with the substrate being aversive and eliciting retreat, similar to the aversive effects of the original release procedure.

Finally, initial outbound orientation at distances between 4.5 m and 16 m on the column would represent points at which the forager is not close to the nest and that when responding to an aversive incident, they can retreat along the column while remaining on the pheromone. This means foragers should orient, not in the outbound of their full vector but to the outbound direction of their current route segment along the column, in order to remain on the pheromone. We see evidence of this orientation to the current route in two conditions: the distant initial headings at 4.5 m and 14 m, where foragers are only oriented to the current outbound route compass direction and not the full outbound vector. In many of the conditions where we observed this retreat, the directional differences between the global outbound vector and the outbound direction of the current route were quite small, thus making differentiating between these two directions difficult using 95% CIs; yet we find multiple instances of orientation only to the current route alone while in no condition do we find orientation to the outbound global vector alone. Coupled with the other instances of inbound route-segment orientation such as in the 3-m paths of distantly displaced column head (24 m) ants, these findings indicate that a mechanism may exists for these foragers to orient in relation to part of their vector and not their full vector state, similar to foragers orienting to their final path segment during backtracking (Freas et al., 2019a; Wystrach et al., 2013) or when orienting via only ocelli (Schwarz et al., 2011). Orientation to vector segments rather than the global vector, in particular during the inbound route, would allow foragers to retrace the non-straight pheromone trail instead of leaving the pheromone to travel in a straight-line to the nest as a shortcut. How this segment-based orientation interacts with the path integration system is interesting and merits further research.

## Conclusions

As highlighted by Cheng’s research (Cheng, 2022; Cheng et al., 2009; Freas & Cheng, 2022; Freas et al., 2019a, b, c; Graham & Cheng, 2009; Islam et al., 2020, 2021, 2022; Schultheiss et al., 2016), research on ant species living in different ecologies has revealed a rich toolkit of navigational mechanisms that function together to produced impressive navigational behaviours. Here we show that *Formica* *obscuripes* attend to both the visual panorama and a path integrator for orientation, with the pheromone’s presence acting as a cross sensory, non-directional verification cue, confirming the correct path and continued orientation to these other strategies. Backtracking behaviour in this species is elicited by a combination of unfamiliar terrestrial cues and the absence of the pheromone, thus operating on a combination of mechanisms observed in both solitarily and socially foraging species. We also characterise a novel form of goalless orientation, an initial retreating behaviour that is modulated by the forager’s path integration system. The behaviour directs disturbed foragers back along their outbound column route for a short distance before recovery, presumably as a defensive response to threat.

### Supplementary Information

Below is the link to the electronic supplementary material.

Supplementary file1 (PDF 448 KB)

## Data Availability

The raw data for all testing conditions is publicly available online at OSF.IO: 10.17605/OSF.IO/MQ4UE

## Declarations

There are no provincial or federal governmental regulations guiding the research of invertebrates in Canada, where testing occurred. Manipulations were non-invasive and tested individuals were marked with acrylic paint (Tamiya^TM^) to prevent re-testing and then returned to the nest upon the conclusion of testing. Subsequently, they were witnessed resuming their foraging behaviour on subsequent days.
