# Supplementary material for: Directed retreat and navigational mechanisms in trail following Formica obscuripes
Source: Learn Behav. 2023 Sep 26;52(1):114–31. doi: 10.3758/s13420-023-00604-1 (PMC10923983; doi:10.3758/s13420-023-00604-1)

Supplemental Table 1. Statistical analysis for the distant displacement conditions. * next to each predicted direction denotes that this direction falls within the condition's 95% confidence interval.

				Rayleigh Test		95% CI		Predicted directions			
								Inbound		Outbound	
Condition		n	Mean vector	*Z*	*p*	Minus	Plus	Global Vector	Route Segment	Global Vector	Route Segment
**Distant initial headings**											
0m (Nest)		21	192.9º	1.32	0.271	-	-	14º	40º	194º	220º
4.5m		20	230.4º	6.11	0.002	201º	260º	14º	40º	194º	*220º
7m		30	175.1º	12.46	< 0.001	156º	195º	14º	40º	*194º	*180º
11m		23	169.6º	5.93	0.002	139º	200º	14º	0º	*194º	*180º
14m		22	170.8º	10.07	< 0.001	150º	192º	14º	0º	194º	*180º
16m		23	172.9º	8	< 0.001	148º	198º	14º	0º	*194º	*187º
24m		20	89.9º	0.49	0.619	-	-	14º	7º	194º	187º
**Distant headings**											
0m (Nest)	at 30cm	15	272.7º	1.04	0.361	-	-	14º	40º	194º	220º
	at 3m	15	200.7º	4.32	0.011	164º	237º	14º	40º	*194º	*220º
4.5m	at 30cm	17	225.5º	8.76	<0.001	203º	248º	14º	40º	194º	*220º
	at 3m	17	213.3º	5.47	0.003	183º	244º	14º	40º	*194º	*220º
7m	at 30cm	15	161.8º	3.97	0.016	123º	201º	14º	40º	*194º	*180º
	at 3m	15	185º	3.35	0.032	142º	228º	14º	40º	*194º	*180º
11m	at 30cm	15	185.2º	8.23	<0.001	161º	210º	14º	0º	*194º	*180º
	at 3m	15	184.3º	7.56	<0.001	158º	211º	14º	0º	*194º	*180º
14m	at 30cm	16	173.2º	3.69	0.022	135º	212º	14º	0º	*194º	*180º
	at 3m	16	355.5º	6.31	0.001	328º	23º	*14º	*0º	194º	180º
16m	at 30cm	15	160.3º	3.24	0.036	116º	204º	14º	0º	*194º	*187º
	at 3m	15	4.0º	7.85	<0.001	338º	30º	*14º	*0º	194º	187º
24m	at 30cm	16	162º	0.57	0.574	-	-	14º	7º	194º	187º
	at 3m	16	4.6º	15.85	<0.001	2º	7º	14º	*7º	194º	187º
**Distant initial headings**											
0m (Nest) On pheromone		34	32.6º	12.23	<0.001	12º	53º	*14º	*40º	194º	220º
14m Un-Mirrored		22	165.6º	14.55	<0.001	150º	181º	14º	0º	194º	*180º
14m Mirrored		21	232.2º	3.1	0.04	276º	189º	14º	0º	*194º	180º

Supplemental Table 2. Statistical analysis for the local displacement conditions. * next to each predicted direction denotes that this direction falls within the condition's 95% confidence interval.

Condition				**Rayleigh Test**		**95% CI**		**Predicted directions**			
								Inbound		Outbound	
		n	Mean Vector	*Z*	*p*	Minus	Plus	Remaining Vector	Current Route	Remaining Vector	Current Route
**On Column Displacements**											
Release into Column											
7m	at 30cm	16	191.6º	3.88	0.018	154º	229º	40º	0º	*220º	*180º
	at 1m	16	359.6º	11.62	<0.001	344º	15º	40º	*0º	220º	180º
14m	at 30cm	16	178.3º	3.49	0.028	138º	218º	17.4º	0º	*197.4º	*180º
	at 1m	16	350.3º	4.75	0.007	317º	23º	*17.4º	*0º	197.4º	180º
Climb Down to Column											
7m	at 30cm	16	350.6º	8.2	<0.001	328º	13º	40º	*0º	220º	180º
	at 1m	16	359.2º	15.8	<0.001	356º	2º	40º	*0º	220º	180º
14m	at 30cm	16	357.1º	2.7	0.065	-	-	17.4º	0º	197.4º	180º
	at 1m	16	357.1º	12.1	< 0.001	342º	12º	17.4º	*0º	197.4º	180º
**Lateral Displacements**											
0m (Nest) On route	at 30cm	12	356.2º	7.5	0.001	331º	21º	40º	*0º	220º	180º
	at 1m	12	358.5º	8.4	< 0.001	337º	20º	40º	*0º	220º	180º
	Exit	12	0.0º	10.9	< 0.001	349º	11º	40º	*0º	220º	180º
0m (Nest) 2m lateral	at 30cm	16	3.3º	3.4	0.032	322º	44º	*40º	*0º	220º	180º
	at 1m	16	24.9º	4.5	0.009	350º	59º	*40º	*0º	220º	180º
	Exit	16	161.9º	3.2	0.04	120º	204º	40º	0º	220º	*180º
7m On route	at 30cm	12	206º	0.2	0.79	-	-	40º	0º	220º	180º
	at 1m	12	357º	11.7	< 0.001	351º	3º	40º	*0º	220º	180º
	Exit	12	2.8º	12	< 0.001	2º	4º	40º	0º	220º	180º
7m 2m lateral	at 30cm	12	42º	1.5	0.219	-	-	40º	0º	220º	180º
	at 1m	12	8.8º	4.8	0.006	336º	41º	*40º	*0º	220º	180º
	Exit	12	160º	3.8	0.019	121º	199º	40º	0º	220º	*180º
**Above column Displacements**											
Wooden Surface											
0m (Nest)		23	322.2º	0.7	0.489	-	-	40º	0º	220º	180º
14m		29	179.7º	4.8	0.007	145º	214º	17.4º	0º	*197.4º	*180º
Soil Surface											
0m (Nest)		18	13.6º	10.9	< 0.001	355º	32º	40º	*0º	220º	180º
14m		20	359.4º	11.2	< 0.001	340º	18º	*17.4º	*0º	197.4º	180º

Supplemental Figure 1. Panoramic Images of the collection and displacement sites along the foraging route. Labels designate the point along the foraging column foragers were collected or the displacement location if it was off the column.

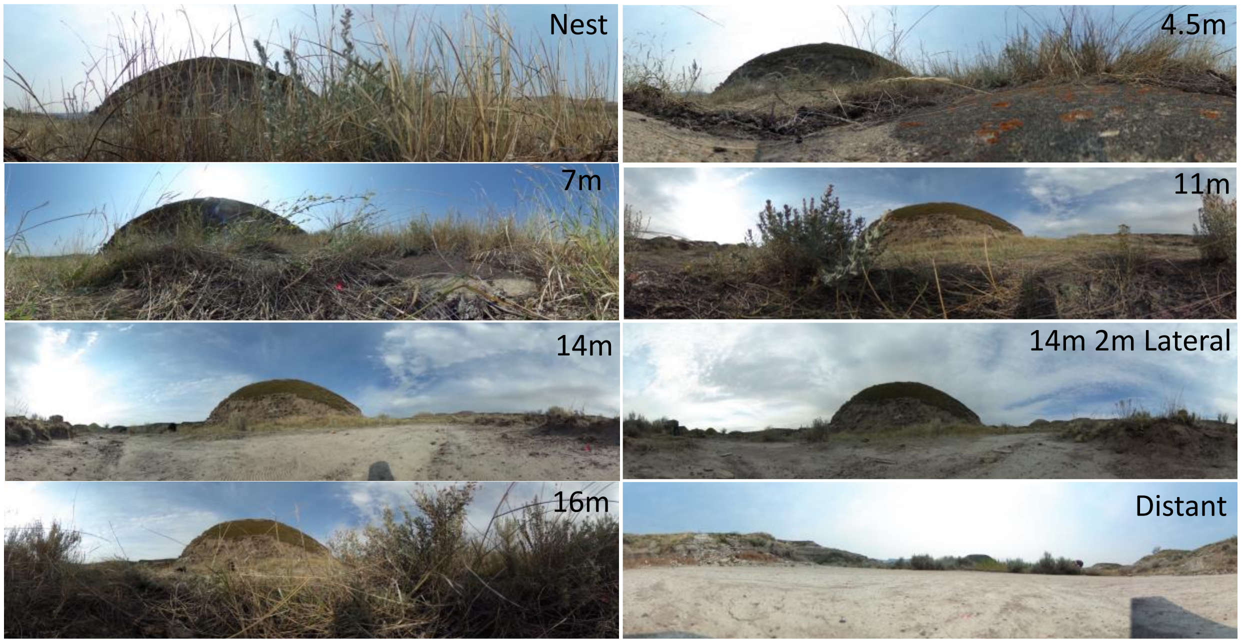

Supplement: Supplementary file 1 — Supplementary file1 (PDF 448 KB) [file 13420_2023_604_MOESM1_ESM.pdf]
